# Nuclear Molecular and Theranostic Imaging for Differentiated Thyroid Cancer

**DOI:** 10.4274/2017.26.suppl.06

**Published:** 2017-01-09

**Authors:** Arif Sheikh, Berna Polack, Yvette Rodriguez, Russ Kuker

**Affiliations:** 1 Columbia University Medical Center, Clinic of Radiology, New York, USA; 2 Dokuz Eylül University Faculty of Medicine, Department of Nuclear Medicine, İzmir, Turkey; 3 Florida International University, Herbert Wertheim College of Medicine, Department of Surgery, Miami, USA; 4 University of Miami Miller School of Medicine, Department of Radiology, Miami, USA

**Keywords:** nuclear medicine, RAI, I-131, I-123, I-124, FDG-PET/CT, thyroid carcinoma, differentiated thyroid cancer, theranostics

## Abstract

Traditional nuclear medicine is rapidly being transformed by the evolving concepts in molecular imaging and theranostics. The utility of new approaches in differentiated thyroid cancer (DTC) diagnostics and therapy has not been fully appreciated. The clinical information, relevant to disease management and patient care, obtained by scintigraphy is still being underestimated. There has been a trend towards moving away from the use of radioactive iodine (RAI) imaging in the management of the disease. This paradigm shift is supported by the 2015 American Thyroid Association Guidelines (1). A more systematic and comprehensive understanding of disease pathophysiology and imaging methodologies is needed for optimal utilization of different imaging modalities in the management of DTC. There have been significant developments in radiotracer and imaging technology, clinically proven to contribute to the understanding of tumor biology and the clinical assessment of patients with DTC. The research and development in the field continues to evolve, with expected emergence of many novel diagnostic and therapeutic techniques. The role for nuclear imaging applications will continue to evolve and be reconfigured in the changing paradigm. This article aims to review the clinical uses and controversies surrounding the use of scintigraphy, and the information it can provide in assisting in the management and treatment of DTC.

## Abstract (tr)

Geleneksel nükleer tıp, moleküler görüntüleme ve teranostik kavramları ile hızla değişmektedir. Ancak bu prosedürlerin değeri diferansiye tiroid kanserleri (DTK) alanında küçümsenmektedir. Bu durum kısmen hastalığa klinik yaklaşımdaki gelişmelere kısmen de klinisyen ve nükleer görüntüleyicilerin bu görüntüleme ile ilgili bilgilerinin eksikliği nedeniyle değerinin anlaşılamamasına bağlıdır. Sonuç olarak, DTK’da sintigrafinin kullanımı açısından veriler ancak sınırlı bir yarar göstermiş, ve yakın zamanda yayımlanan Amerikan Tiroid Birliği yönergesinde (1) de ifade edildiği gibi hastalığın tedavisinde görüntüleme kullanımından uzaklaşma eğilimi doğmuştur. Her ne kadar görüntülemenin değeri tartışmalı olsa da, görüntülemeye ve bunların DTK yönetiminde uygulanmasına daha sofistike bir yaklaşımın gerekliliği ve yararı konusunda evrensel görüş birliği vardır. Radyo-farmasötikler ve teknolojide, DTK nükleer görüntülemesinde yararı kanıtlanan birçok gelişme olmuştur ve bu gelişmeler devam etmektedir. Gelecekte yeni terapötik ve görüntüleme seçeneklerinin ortaya çıkmasıyla birlikte, görüntülemenin yararı yeniden revize edilebilir. Bu makale sintigrafinin kullanımının klinik faydaları ve etrafındaki tartışmalarla birlikte, sağladığı bilgilerin DTK yönetimi ve tedavisindeki etkilerini araştırmaktadır.

## INTRODUCTION

## Clinical Radioiodine Imaging

Over the years, several options for imaging differentiated thyroid cancer (DTC) in nuclear medicine have been introduced, allowing for the progression in both the tracers and technology used. The standard imaging for DTC has been radioactive iodine (RAI), in particular iodine-131 (I-131). In fact, this can be considered the first truly theranostic agent as it provides both diagnostic capabilities and therapeutic abilities. It is a high energy tracer with a half-life of about 8 days, obtained from byproducts of nuclear reactors, and thus is the cheapest form of RAI. It can be used in pre-therapy imaging, but is also the sole radiopharmaceutical for post-therapy imaging, making it an ideal theranostic agent. The drawbacks against I-131 imaging are its lower resolution and higher radiation exposure profile as compared to other radiopharmaceuticals. In pre-therapy imaging, this leads to lower accuracy of disease detection.

Iodine-123 (I-123), since its introduction in nuclear medicine, has gained popularity in pre-therapy imaging. Its lower imaging energy peak allows for improved resolution as well as lower radiation exposure to the patient. This, in turn, leads to improved sensitivity for disease detection as compared to pre-therapy I-131 imaging as well as better predictive value of the post-therapy scan than the pre-therapy I-131 scan (2). Unlike I-131, it is cyclotron produced and has a shorter half-life of approximately 13 hours. The cyclotron production makes I-123 much more difficult to produce and distribute, and as such, a more expensive tracer. Also, due to the lack of cyclotron facilities, especially in third world countries, its availability may be severely limited. Its shorter half-life realistically limits imaging to within 48 hours; therefore, lesions with low grade uptake may not show up on delayed images. Thus, performing extended dosimetry is practically not feasible with I-123.

There are certain advantages of I-131 over I-123. The usage of I-131 may be more effective in obese patients due to better tissue penetration. Its long half-life allows for delayed imaging, which could improve image contrast over time and increase tumor accumulation, resulting in better visualization of the disease including portions that might have been missed on images taken at earlier time points. This could lead to better therapy planning assessment. Moreover, I-131 is well suited for dosimetry should one wish to perform the procedure. Although I-123 is considered superior in many instances in the pre-therapy evaluation, I-131 still remains the standard and currently the only possible modality for post-therapy imaging.

## SPECT and CT Imaging

On the technological side, advancements in RAI imaging relate to the development of single photon emission computed tomography-computed tomography (SPECT-CT), which have dramatically improved the capabilities for disease evaluation and management. Generally, RAI imaging is performed as a whole body planar scan. Additional static dedicated views of the neck and chest regions are usually recommended in order to evaluate for potential artifacts, as well as to increase disease detection sensitivity. Pinhole imaging is sometimes used with these views as it may improve resolution and sensitivity, but at the cost of geometrically distorting the detected lesions. Following the introduction of SPECT, it was recognized that the 3-dimensional views it provided could further enhance disease evaluation. RAI images inherently have several artifacts and thus lesions can be difficult to localize without further more definitive anatomic information being available (3).

SPECT-CT is a device that sequentially combines the ability of a camera to perform RAI imaging by SPECT, as well as a clinical CT scan, all performed within one imaging session. The two images acquired can then be fused, resulting in a sound anatomic correlation of uptake localizations detected by the SPECT study. Nevertheless, its routine use is often hindered by the perception that the additional CT component significantly increases the radiation burden to the patient. What is often overlooked by nuclear medicine physicians and referring clinicians is that the CT portion of the study is actually first for image correction (attenuation, scatter, etc.) that enhances the SPECT portion of the study, and that this procedure is not simply an overlay of the two studies. This combination of SPECT enhancement and definitive anatomic localization leads to improvements in disease detection and demonstrable changes in the management of pre-therapy, post-therapy and follow-up scans (4,5). Another misunderstanding amongst referring physicians is that the CT portion of the study is usually a low quality study since its primary purpose is for image correction and anatomic localization, rather than a full diagnostic capability. Whereas many current SPECT-CT devices have multidetector CT capabilities that can perform clinical diagnostic imaging, they are usually only used within limited capability. Thus, any extra radiation burden to the patient from SPECT-CT diagnostic imaging is less significant than perceived, while markedly enhancing management capabilities of the patient’s disease.

## Post-Therapy Imaging: The ‘True’ Diagnostic Scan

**Figure 1:**
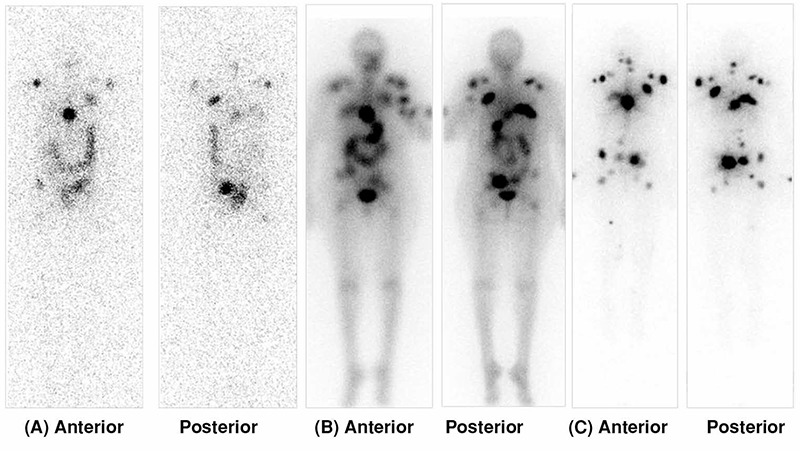
Images show (A) Whole Body Pre-therapy (dosimetry) scan 3 days following administration of 148 MBq I-131. Post-therapy scan 1 day (B) and 8 days (C) following administration of a therapeutic amount of I-131. There are clearly more lesions seen in the post-therapy scan than the pre-therapy one, but the timing of the scan is also important, as (C) shows more lesions than does (B). As noted by Sabra, et al. (6) the appearance of lesions not seen in the pre-therapy scan portends a lower therapeutic potential for those seen only in the post-therapy scan, and thus an early identifier of potential treatment failure, well before the first post-treatment Thyroglobulin levels are drawn. This could not have been known if the Pre-therapy scan was not performed

The post-therapy scan is performed after administration of therapeutic activities of I-131, generally >1.22 GBq. It is well recognized that the post-therapy scan is the most sensitive scan for the detection of RAI avid disease, and thus can be considered as the gold standard for disease detection (Figure 1). In that sense, the post-therapy scan is the optimal single scan for disease detection and staging, and therefore, a paramount diagnostic study. The post-therapy scan provides a wealth of information on patient management when performed correctly, and this data is prudently integrated into the patient’s overall clinical information. The scan provides the greatest information about the extent of disease as well as guidance on predicting treatment response (6), and can lay out the kind of follow-up to be performed after treatment even though it may not alter subsequent therapy administration. Since the count rate is often higher than the rate found in pre-therapy scans, this is accepted as an appropriate time to consider performing additional SPECT-CT views to better understand the location of the disease, especially of lesions not detected on planar whole body images. The recently released American Thyroid Association (ATA) 2015 guidelines “strongly recommend” to perform SPECT-CT scanning after administration of any therapeutic RAI (1).

## Pre-Therapy Imaging

In contrast to post-therapy scanning, the use of the pre-therapy scan remains controversial for several reasons. Although usually referred to as the diagnostic scan, the most often cited reason against its routine use is precisely its limited diagnostic value and poor ability to localize disease, which is clearly inferior to post-therapy scanning. Futhermore, it has little impact on changing management, and the issue of stunning is of great concern as it could actually lead to worse outcomes. As a result, the use of pre-therapy scintigraphy has been declining and not found to be useful by many. Nevertheless, the value that the diagnostic scan does provide is often overlooked. In the correct setting, it could provide a wealth of information that could assist in the approach to RAI therapy, as well as in the overall management of the patient.

## Theranostic Value and Impact on Disease Management

The information provided by the pre-therapy scan has a much broader role in managing patients than simply its staging and diagnostic capabilities alone. Some studies have noted that if attention is paid to the technical aspects when performing a pre-therapy scan, it improves the diagnostic capability of the scan. In particular, performing a scan at a slower speed, spending time to do spot views, and performing extra pinhole images can markedly improve diagnostic capability. As noted earlier, performing SPECT-CT improves diagnostic ability compared to planar imaging alone. Hence, despite the perception that there is little data to support doing pre-therapy scans, there has actually been a number of studies over the years supporting its use (7,8).

The next question is whether these scans alter management. This question is particularly posed for the low or moderate risk patients. Because of the ability to upstage patients, it is important to answer if the frequency of these changes justifies the cost and effort of performing a scan. The supporting studies show management changes in a significant number of patients ranging between 22-42% of the time (9,10). The alterations in management include increasing RAI administered activities due to upstaging patients, increasing or decreasing RAI administered activities based on the residual tissue volume and/or number of lesions seen and warrant additional surgical management not otherwise anticipated. Although less controversial in higher risk patients, one may be able to manage patients in that setting as well if it is thought that therapeutic approaches will not change. In some of these patients, dosimetry could be considered as a pre-therapy evaluation to guide treatment and disease management, but there is little data to support its use. Nevertheless, the information provided by such an evaluation and its impact could outweigh any negative effect the procedure may have due to stunning or cost.

The final question then arises as to whether these differences in management lead to improvement in outcomes. This last issue is more difficult to answer because the kind of management changes proposed by studies have become less relevant in more updated guidelines, which have become more conservative in their approaches to surgery, RAI administration, as well as follow-up management. Thus previous studies which reported changes in management based on pre-therapy scans may have less impact according to more recent guidelines where the resultant change is no longer found to have a clinical impact. These newer management strategies themselves remain contentious and old data has to be continuously re-visited or re-studied in order to support the ongoing relevance of RAI pre-therapy scans in the era of novel management guidelines. Nevertheless, recent studies continue to show an impact, still lending support that the procedure is useful when executed correctly (11).

## Additional Utility of Pre-Therapy Imaging

**Figure 2:**
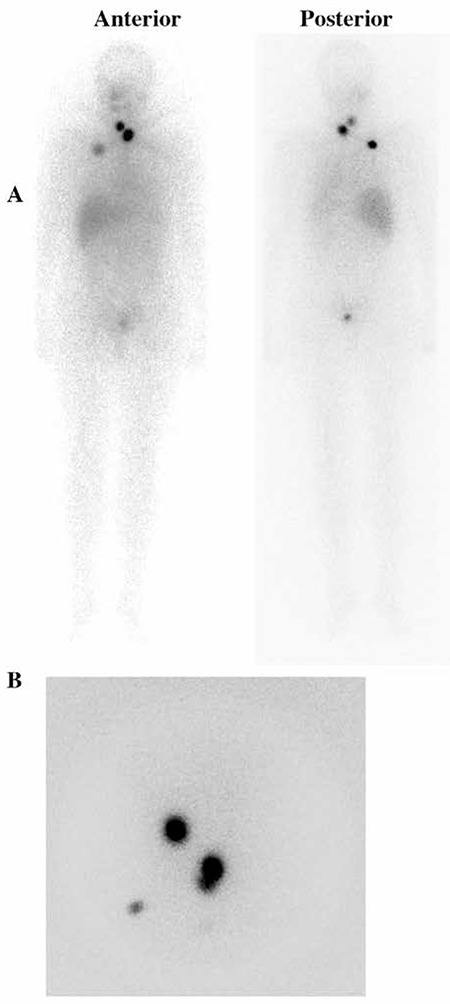
(A) Post-treatment whole body scan showing residual neck thyroid tissue and lung metastatic disease, (B) Pinhole neck images show an additional cervical lymph node not appreciated on the whole body scan.

The pre-therapy scan may provide additional information that could be helpful in clinical care and treatment decisions for patients. In a study focused on the overall utility of pretreatment imaging, Van Nostrand et al. (12), investigated not only increased diagnostic utility, but also other clinical management issues. These included regulating salivary gland RAI uptake, to reduce sialadentis by administration of sailogogues, and minimizing the absorbed dose to the colon by administration of laxatives if excessive bowel retention was seen, or changing post-treatment radiation safety precautions if overall clearance was found to be significantly different than what was empirically calculated (Figure 2). They noted that the scans could provide clinically relevant information in 53% of patients (Table 1). Additional studies have also shown that visualization on a pre-therapy scan, or the visualization of lesions as compared to the post-therapy scans, are prognostic in the ability of a lesion to respond to therapy (13). The pattern of lesional uptake is perhaps not so detailed to propose the scan as a true diagnostic agent in this setting. Therefore the term “diagnostic scan” is more appropriate for the post-therapy setting. The pre-therapy RAI scan should be more appropriately referred to as a “management planning scan”.

## The Stunning Phenomenon

DTC is a unique disease in that the use of imaging itself can impact therapeutic efficacy, and thus must be used judiciously. The use of intravenous CT contrast prior to RAI imaging and subsequent therapy could blunt I-131 uptake and therapeutic outcomes; therefore, it is recommended to wait at least a month between the contrast administration and the RAI procedures (1). In addition, the use of RAI scanning itself is subject to the stunning phenomenon. Stunning is the event in which the uptake seen in a post-therapy scan in certain tissues of thyroid origin appears to have less uptake than predicted by the pre-therapy scan. Although the phenomenon of stunning has been universally observed, its subsequent clinical impact remains controversial. Some studies have shown that pre-therapy scans subsequently lead to a decrease in the success of ablations (14), although several other studies have not shown such results (15,16) including those in high risk patients (17). Studies at the cellular level demonstrate that stunning is a real problem, particularly with I-131 (18), although the reason is likely to be multifactorial (19). Due to the stunning concern, I-123 has gained interest, potentially to replace I-131, both for its apparent increased accuracy at disease detection, and to avoid stunning. Interestingly, some studies have shown that even I-123 can result in stunning, albeit with lower frequency than I-131 (20) or in similar amounts. Other reports may suggest that stunning may not be a clinically relevant phenomenon (21). It has been recommended that RAI therapy be administered within two days of the pre-therapy scan to minimize the clinical impact of the stunning phenomenon. Standard dosimetry is performed using I-131, thus, the issue of stunning has to be taken into account.

To minimize the impact of stunning on subsequent treatment, several other strategies can be utilized. Traditionally, pre-therapy scans have been done with a standard administered activity of I-131. However, factually, post-thyroidectomy patients may have variable amounts of remnant volume. The absorbed dose in the remnants, thus, could be variable. In remnants receiving higher absorbed doses, stunning may be a true clinical issue. When a large remnant volume is suspected or anticipated in a patient with recent thyroidectomy, a screening pertechnetate scan or neck ultrasound could be utilized to determine the volume of the remnant tissue. The administered activity of I-131 can be modified based on the residual amount of tissue to decrease stunning effect. If a significantly large remnant volume is identified, a completion thyroidectomy may be considered.

In the final analysis, it should be noted that the stunning phenomenon has been demonstrated to be a physiologic event based on studies which have evaluated the efficacy of thyroid tissue ablation as an endpoint. However, studies that have looked at the overall disease response or long term outcomes have not found significant impact of this phenomenon (22).

## F-18 FDG PET/CT Imaging in Well-Differentiated Thyroid Cancer

Although RAI imaging is the standard for DTC, and can be optimized for higher diagnostic yield and clinical utility, there is clearly a group of tumors with poor differentiation or classified as “differentiated” by morphology, which are not RAI avid. These tumors cannot be imaged by RAI. There have been attempts to image these lesions with Thallium-201, Tc-99m Sestamibi and others; however, with the advent of F-18 flurodeoxyglucose (FDG) and positron emission tomography (PET), a strong imaging tool was added to our armamentarium for disease management. F-18 FDG PET/CT has gained wide acceptance for the detection, staging, and management of a multitude of malignant tumors. The role of F-18 FDG PET/CT in well-differentiated thyroid cancer (WDTC) is limited since increased FDG uptake is mostly restricted to aggressive and undifferentiated, high grade tumors, with no significant FDG uptake in well- differentiated thyroid tumors (23). In the 2015 ATA guidelines, F-18 FDG PET/CT is strongly recommended in high risk WDTC patients with elevated serum thyroglobulin (Tg) (generally >10 ng/ml), and negative radioiodine imaging (1). According to the ATA guidelines, F-18 FDG PET/CT may also be considered a) as part of initial staging in poorly differentiated thyroid cancers and invasive Hurtle cell carcinoma, especially those with other evidence of disease, found on imaging or due to elevated serum Tg levels, b) as a prognostic tool in patients with metastatic disease in order to identify lesions as well as patients at highest risk of rapid disease progression and disease-specific mortality, and c) as an evaluation of post-treatment response, following systemic or local therapy of either metastatic or locally invasive disease. Although the utility of F-18 FDG PET/CT is limited in WDTC, the indications for FDG PET/CT depend on the clinical setting and thyroid cancer histology.

## F-18 FDG PET/CT in Thyroid Incidentalomas

A discrete focus of uptake in the thyroid gland by F-18 FDG PET/CT in a patient studied for non-thyroidal purposes is an incidentaloma. The normal thyroid tissue shows no or minimal uptake of FDG. While most thyroid incidentalomas are benign, some are malignant, and can either be primary thyroid tumors or secondary lesions from other tumors. Diffuse FDG uptake generally indicates chronic or acute thyroiditis, however, focal FDG uptake in the thyroid gland indicates a significant risk of malignancy. In a meta-analysis study, Treglia et al. (24), reported that the pooled prevalence and malignancy risk of focal incidental FDG uptake in the thyroid were 1.92% [95% confidence interval (CI): 1.87-1.99%] and 36.2% (95% CI: 33.8-38.6%) respectively, without significant differences among various geographic areas. The most frequent malignant histological type responsible for FDG positive thyroid incidentalomas was papillary thyroid cancer. There was no safe, definite cutoff SUVmax value reported for differentiating malignant incidentalomas from benign ones. However, it was noted that a higher SUVmax value increased the likelihood of malignancy (25). Further ultrasonography and histological investigation is recommended in FDG positive incidentalomas since approximately one third of focal FDG avid thyroid lesions are malignant. ATA guidelines strongly recommend fine needle aspiration cytology (FNAC) for the thyroid nodules which are sonographically confirmed and have demonstrated focal FDG uptake.

## F-18 FDG PET/CT in Thyroid Nodules with Indeterminate Fine-Needle Aspiration Cytology

An indeterminate fine-needle aspiration cytology (FNAC) result of a thyroid nodule is a clinical problem, because 20 to 30% of patients suffer from malignancy. The role of F-18 FDG PET/CT in thyroid nodules with indeterminate FNAC, has been studied to identify the patients who would benefit from lobectomy or thyroidectomy. In a meta-analysis of six studies which investigated the diagnostic role of FDG PET scans in thyroid nodules with indeterminate FNAC, Vriens et al. (26), reported that the pooled prevalence of malignancy was 26%. Pooled sensitivity, specificity, positive predictive value, negative predictive value (NPV), and accuracy of FDG PET were 95% (95% CI, 86%-99%), 48% (95% CI, 40%-56%), 39% (95% CI, 31%-47%), 96% (95% CI, 90%-99%), and 60% (95% CI, 53%-67%), respectively. Sensitivity increased to 100% for the 164 lesions that measured >15 mm in greatest dimension. A negative FDG PET scan ruled out thyroid cancer in patients with >15 mm thyroid nodules and indeterminate FNAC in a pooled population of 225 patients. However, a positive FDG PET did not necessarily indicate malignancy. In another meta-analysis, Wang et al. (27), analyzed seven studies using 267 patients and they reported that the pooled sensitivity and specificity of FDG PET or PET/CT for cancer detection in thyroid nodules with indeterminate FNAC results were 89% and 55%, respectively. These studies clearly demonstrate that the sensitivity of FDG PET or PET/CT is high, but specificity and positive predictive values are very low in differentiating cancer in indeterminate thyroid nodules. In the ATA guidelines, FDG PET/CT is not routinely recommended for the evaluation of thyroid nodules with indeterminate FNAC.

## F-18 FDG PET/CT in Patients with Elevated Thyroglobulin Levels, But Negative Neck Ultrasound and Radioiodine Whole Body Scan

A major clinical challenge in the management of WDTC, is the particular patient that displays elevated Tg but both negative neck ultrasound (US) and I-131 whole body scan (WBS) after initial I-131 treatment. In many cases, the initial high Tg levels gradually decrease over time after I-131 treatment. The decrease in Tg to the lowest level may take months to years when left untreated. However, if Tg antibody is negative and there is no evidence of residual thyroid tissue, a rising level of stimulated or un-stimulated serum Tg over time may indicate which patients have residual or recurrent disease. In this particular group of patients, the most common finding would be local recurrence in the thyroid bed as well as either cervical or mediastinal lymph node metastasis. If the US and I-131 WBS are negative for recurrence and metastasis but Tg is high and rising, two possible reasons can be considered:

1) The tumor or metastatic tissue is too small to be detected with both imaging modalities, or;

2) The tumor might not be able to concentrate iodine but is still able to produce Tg (28).

Cervical US is a very sensitive imaging modality used to detect recurrence in the thyroid bed as well as nodal metastasis in the neck, but it has low specificity in patients with altered anatomy after surgery because of difficulty in differentiating between scar tissue and local recurrence, as well as differentiating between nonspecific nodal growth and nodal metastases. Cervical US also cannot show distant metastases (29,30). If cervical US is negative or equivocal, CT and magnetic resonance imaging may be other useful tools for detecting recurrence and metastases in the neck or mediastinum. If cervical US and I-131 WBS are not able to show recurrence or metastatic disease, F-18 FDG PET/CT can be used. In a recently performed meta-analysis of 20 studies, Caetano et al. (31), reported both the sensitivity and specificity of conventional PET and PET/CT in detecting recurrence of WDTC in patients with high Tg but negative I-131 WBS as 84% and 84% respectively and 93% and 81% respectively. The overall accuracies were 91% and 93% for PET and PET/CT, respectively. Another meta-analysis of 25 studies with 789 patients showed that FDG PET/CT is a more sensitive imaging method than FDG PET in the follow-up of WDTC recurrence or metastases in patients with negative I-131 WBS (32). The increased FDG uptake in iodine-negative recurrent and metastatic lesions could be due to the growth of more aggressive tumor cells that have lost the expression of the membrane glycoprotein sodium-iodide symporter, which is responsible for the active transport of iodine in the thyroid tissue, with increased expression of the glucose transporter-1 (GLUT-1) (31). The F-18 FDG PET/CT may lead to a change in treatment plan in 9 to 54% of cases; especially by detecting unexpected distant metastases.

In the ATA guidelines (1), FDG PET/CT imaging is strongly recommended in patients with negative I-131 WBS and serum Tg >10 ng/ml. Although serum Tg levels correlate with tumor load, and the sensitivity of FDG PET/CT in detecting lesions increases as Tg levels increase, several studies report that FDG PET/CT could also be helpful in selected cases with Tg levels below 10 ng/ml. Shammas et al. (33), reported that the sensitivity of FDG PET/CT in detecting recurrent and metastatic lesions in patients with elevated Tg but negative I-131 WBS changed according to the serum Tg levels of the patients. It was reported that the overall sensitivity, specificity, and accuracy of FDG PET/CT were 68.4%, 82.4% and 73.8%, respectively within the study. The sensitivity of FDG PET/CT at serum Tg levels of less than 5, 5-10, and greater than 10 ng/ml were 60%, 63%, and 72%, respectively. FDG PET/CT changed clinical management in 44% of patients in this study. Another study by Giovanella et al. (34), showed that FDG PET/CT scans were true-positive in 46% of patients who had Tg serum levels <10 ng/ml.

Another issue to be addressed is the effect of TSH stimulation on the sensitivity of FDG PET/CT in detecting recurrent and metastatic disease in patients with negative cervical US and I-131 WBS but high Tg serum levels. Studies have shown that TSH stimulates the glucose transport system by enhancing the number of functional GLUT transporters in the plasma membrane (35). A meta-analysis by Ma et al. (36), revealed the results of 7 prospectively controlled clinical trials which were aimed to establish the effects of TSH stimulation on the uptake of FDG in 168 patients who had elevated serum Tg levels but negative I-131 WBS. The number of patients with FDG true-positive lesions, FDG-detected lesions, and the tumor-to-background ratios significantly increased after TSH stimulation. FDG PET/CT undertaken with TSH stimulation altered clinical management in 9% of patients within five paired studies. TSH stimulation can be done by either T4 withdrawal or recombinant human TSH (rhTSH) administration. In rhTSH stimulation, which avoids the adverse effect of hypothyroidism on quality of life, FDG PET/CT scans can be performed either 24 or 48 hours after rhTSH administration. Leboulleux et al. (37), analyzed 108 lesions and reported that rhTSH stimulated FDG PET/CT was significantly more sensitive than basal PET/CT for lesion detection and tended to be more sensitive in the detection of involved organs. Basal FDG PET/CT altered clinical management in 19% of patients, whereas lesions found only by rhTSH stimulated FDG PET/CT contributed to an altered therapeutic plan in eight patients among whom only 6% were true positive on pathology. According to their results, rhTSH stimulated FDG PET/CT significantly increased the number of lesions detected, but the numbers of patients in whom any lesion was detected did not differ between basal and rhTSH stimulated PET/CT scans.

**Figure 3:**
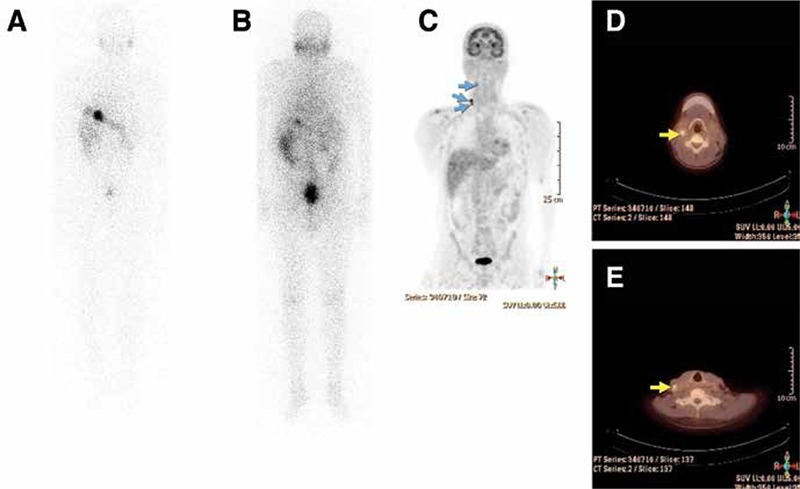
A 65 year-old patient with papillary thyroid cancer with follicular variant. Nine months after initial I-131 treatment, a diagnostic I-131 whole body scan was performed (A, anterior and B, posterior) with no evidence of residual or recurrent/metastatic disease. Neck ultrasound did not show any pathological findings. However, the patient had a high and rising serum Tg level suspicious for metastatic disease. FDG PET/CT imaging showed multiple lymph node metastases (white and blue arrows) in the right neck (C). Cervical lymph node dissection was performed and metastatic lymph nodes were resected (D and E)

Figure 3 shows a 65-year-old patient with papillary thyroid cancer follicular variant. The patient had undergone a total thyroidectomy. A multifocal papillary cancer, the largest tumor being 2 cm in diameter and one lymph node metastasis, was found on histopathological examination. Nine months after 150 mCi radioiodine ablation therapy, I-131 WBS and neck US were normal. Stimulated Tg was 10.1 ng/ml and Anti-Tg was normal. FDG PET/CT imaging was performed to search for recurrent/metastatic disease. Cervical metastatic lymph nodes were detected with FDG PET/CT in multiple levels of the patient’s right neck, which were confirmed by histological examination.

## The Value of F-18 FDG PET/CT in Determining Prognosis

FDG PET/CT can be used as a prognostic tool for patients with DTC. A poor prognosis and reduced survival can be predicted in patients who have metastatic DTC and high FDG uptake, which suggests dedifferentiated, aggressive, and metabolically active tumor cells. Wang et al. (38), investigated the prognostic value of FDG PET/CT in 125 patients with high Tg and negative I-131 WBS. In this study, univariate analysis demonstrated that survival was reduced in patients over 45 years of age, those with distant metastases, PET positivity, high rates of FDG uptake, and high volume of FDG-avid disease (>125 mL); multivariate analysis demonstrated that the single strongest predictor of survival was the volume of FDG-avid disease. Masson-Deshayes et al. (39), evaluated quantitative FDG PET/CT parameters such as the number of FDG-avid lesions, the SUVmax, the SULpeak of the lesion with the highest FDG uptake, the overall metabolic tumor volume (MTV), and the total lesion glycolysis (TLG), in predicting progression-free and overall survival in 35 patients with metastatic DTC. Progression-free survival was better in patients with less than 10 FDG-avid lesions, a SUVmax less than 10, a SULpeak less than 5, and a TLG less than 154. In their study, cox analysis displayed that only the PET scan result was predictive of survival (age, tumor node metastasis (TNM) stage, histology, and the I-131 WBS were not associated with prognosis). In the univariate analysis, prognostic factors for progression-free survival and overall survival were the SUVmax, the SULpeak, and the TLG. The number of FDG-avid lesions was significantly associated with progression-free survival, but not the MTV. In the multivariate analysis, the number of FDG-avid lesions and the SULpeak were independent prognostic factors. These studies showed that high FDG uptake in lesions may be a strong indicator of patient prognosis, and that management can be altered according to the results of FDG PET/CT findings.

## F-18 FDG PET/CT in Initial Staging or Follow-up in High Risk Patients with Aggressive Histological Subtypes

Hurtle cell carcinoma (HTCC), poorly differentiated thyroid carcinoma, and anaplastic thyroid carcinoma are not common thyroidal malignancies. These tumors usually do not concentrate a significant amount of radioiodine and their prognosis is worse than WDTC. FDG PET/CT can be used in the initial diagnosis to evaluate the extent of the disease and to obtain prognostic information (40). HTCC, about 3.6% of thyroid cancers, used to be considered as a subtype of WDTC, but because of its biological behavior, has been accepted as an aggressive histologic subtype. Few studies have been published so far on the role of FDG PET in the management of HTCC patients. The sensitivity and specificity of FDG PET in HTCC have been reported as 92 to 95% and 80 to 95%, respectively. According to the ATA guidelines (1) FDG PET/CT may be considered in the initial staging of poorly differentiated thyroid cancers or HTCC, especially those with evidence of disease on other imaging modalities or with elevated serum Tg levels.

Nascimento et al. (41), found that FDG PET/CT was more sensitive than I-131 post-ablation WBS in detecting individual lesions in patients with aggressive histology thyroid cancer, mostly tall cell papillary carcinoma and poorly differentiated carcinoma. Both FDG PET/CT and post-ablation WBS were complementary with 41% of the lesions detected only by FDG-PET/CT and 31% only by post-ablation WBS. The authors reported that the only risk factor for abnormal FDG-PET/CT was a stimulated Tg level measured at ablation >10  ng/mL, with persistent disease showing FDG uptake in 72% of the patients with a stimulated Tg >10 ng/mL and in 10% of the patients with a stimulated Tg ≤10 ng/mL.

Rosenbaum-Krumme et al. (42), analyzed the benefit of FDG PET/CT at initial diagnosis in 90 patients with high-risk differentiated thyroid carcinoma and determined whether the FDG PET/CT results led to a deviation from the standard procedure. TNM staging changed due to the FDG PET/CT results in 8 patients, and clinical management changed in 19 of the 90 patients (21%), including all patients with only FDG-positive lesions and all patients with both FDG-positive and iodine-positive lesions. The same authors recently published three year follow-up results of their initial series (43). In their analysis of 109 patients with high risk DTC, the NPV of FDG PET/CT scans at initial staging, regarding full remission on follow-up, were 85% and 91% for patients without FDG-positive lesions and for patients without any lesions, respectively. They concluded that the change in management in patients with iodine-negative lesions can lead to a higher rate of full remissions during follow-up after additional surgery. Therefore, FDG-PET/CT can be considered in all high-risk DTC patients in the context of the first radioiodine therapy to improve patient management and risk stratification.

**Figure 4.1:**
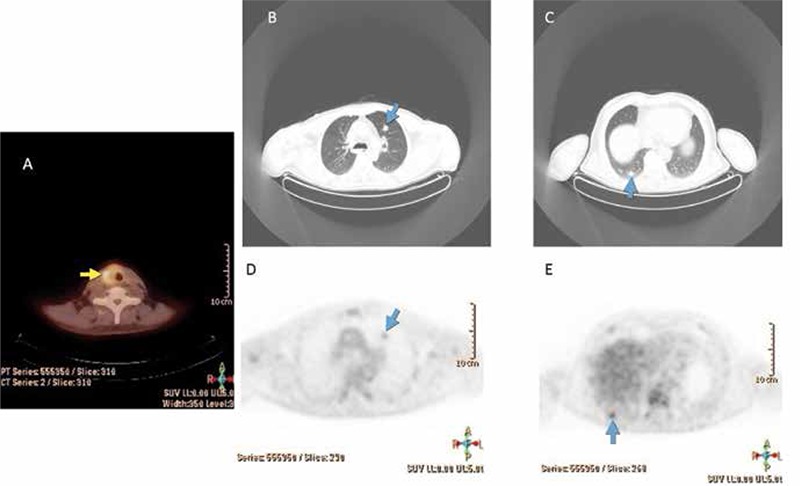
F-18 FDG PET/CT images of a high risk patient with papillary thyroid cancer with folicular variant. Increased FDG uptake was seen in a right thyroid lobe mass invading adjacent soft tissue structures (A, yellow arrow). There are also left and right small lung nodules which show increased FDG uptake (B, C, D, E, blue arrows)

**Figure 4.2:**
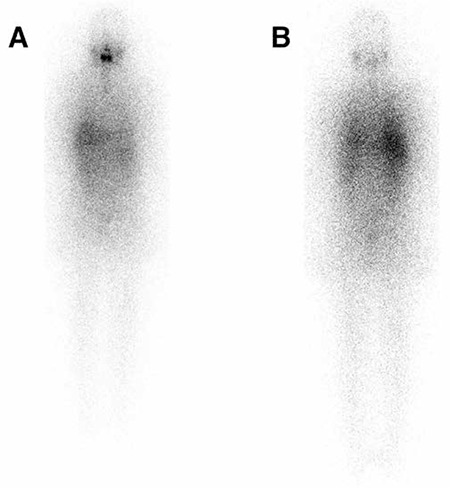
Post-therapy I-131 anterior (A) and posterior (B) whole body images of the same patient which show no evidence of RAI avid metastatic disease.

Figure 4.1 shows FDG PET/CT findings in the initial staging of a high risk patient with papillary thryoid cancer with follicular variant. The patient had a large mass in the right thyroid lobe invading peripheral soft tissues and two lung nodules (one on the right, one on the left) with the greatest diameter of 1 cm which were suspicious for metastases. FDG PET/CT showed remarkably increased FDG uptake in the primary thyroid tumor and mild to moderate uptake in both lung nodules which were consistent with metastases. One month after FDG PET/CT, total thyroidectomy, with laryngectomy and right lateral neck dissection was performed and histopathological examination demonstrated thyroid papillary carcinoma with follicular variant, laryngeal involvement and metastatic lymph nodes at level 4. Two months after the surgery, the patient received 200 mCi of I-131. Post-treatment WBS (stimulated Tg: 42 ng/ml) did not reveal any residual or metastatic I-131 uptake in the whole body images (Figure 4.2). The lung nodules did not show radioiodine uptake. On follow-up, both Tg and the number of metastatic nodules in the lungs increased which confirmed the FDG PET/CT findings. The patient was accepted as having radioiodine refractory disease.

## F-18 FDG PET/CT in Management and Therapy Evaluation of Patients with Radioiodine Refractory Disease

Most patients with RAI-refractory disease (disease which is not responsive to RAI treatment) can be categorized in 4 groups:

1) Patients with metastatic disease that does not show RAI uptake at the time of initial treatment,

2) Patients whose tumors lose the ability to uptake RAI after previous evidence of uptake,

3) Patients with retained RAI uptake in some lesions but not all and,

4) Patients with metastatic disease that progresses despite RAI uptake in the lesions (44).

Prognosis and survival in patients with RAI-refractory disease are variable; some patients show rapid progression of the disease, while some show indolent disease. Currently, novel treatment options for these patients are being clinically evaluated. Multi-targeted thyrosine kinase inhibitors (TKIs), MAPK pathway inhibitors, and angiogenesis inhibitors, are some of these treatments. TKIs are limited to a highly select group of metastatic patients, because their toxicities are sometimes serious and can be fatal (45). FDG PET/CT may have a role in selecting these patients and evaluating the early response to TKI agents. There are studies investigating the efficacy of FDG PET or PET/CT in the early treatment response evaluation of lesions that were FDG positive at baseline (46,47). Carr et al. (47), conducted a phase II study to assess the efficacy of sunitinib in patients with RAI-refractory disease. They designed their study using at least one FDG PET/CT avid lesion with uptake clearly above the blood-pool background, an objective criterion for trial entry, and evaluated the response per FDG PET/CT after 7 days of treatment as an early indicator of response. The disease control was in 78% of patients with a significant average SUV percent change in Response Evaluation Criteria In Solid Tumors (RECIST) response. The average SUVs significantly decreased in patients with partial/complete response and stable disease as compared to the values in patients with progressive disease. However, Kloos et al. (48), did not find any clear correlation between FDG PET response (the % change in SUV_max_ and metabolic volume as compared to pretherapy values) and objective tumor response. They also did not report a consistent change in SUV_max_ and MTV pattern. It is clear that more studies are needed to investigate the specific role of PET/CT in patients with RAI-refractory disease.

## F-18 FDG PET/CT for Radioguided Surgery

Radioguided surgery (RGS) with F-18 FDG has been used in malignancies like colorectal carcinoma, malignant melanoma, breast carcinoma and metastatic head and neck carcinoma. In this technique, a handheld PET probe has been used to locate FDG-positive loco-regional metastases. A limited number of studies have been published on RGS with FDG in thyroid cancer patients with FDG positive and I-131 negative lesions (49,50,51). It has been reported that a hand-held probe could be complementary to FDG PET/CT imaging, in intraoperative detection of FDG positive lesions. In these studies, the intraoperative PET probe did not identify any new foci during surgery, however it localized all the lesions which were visualized on PET/CT images and thus enabled the surgeon to verify that all the detected foci had been completely resected.

## F-18 FDG PET/CT for External-Beam Radiotherapy Planning

External-beam radiotherapy (EBRT) is a rarely used therapy modality in WDTC patients. However, EBRT could be useful in high risk patients who have gross residual non-RAI-avid locoregional disease remaining after surgical resection and/or metastatic lesions. These tumors are more likely to be more aggressive and FDG positive on PET/CT imaging. EBRT can be considered as an adjuvant therapy for mostly older patients (>45 to 50 years) who have undergone complete surgical resection of all visible non-RAI-avid tumor in the setting of gross extrathyroidal extension into surrounding major structures. ERBT is not routinely recommended as an adjuvant therapy for low risk disease, for patients with microscopic extrathyroidal extension detected only on histological examination, or patients with locoregional lymph node involvement in the absence of other very high risk features (52).

The main objective of EBRT treatment is to deliver the highest dose of radiation possible to the tumor without causing damage to the surrounding normal tissues. Image-guided-intensity modulated radiation therapy [IG-IMRT or image-guided radiotherapy (IGRT)] can be a valuable radiotherapy technique in order to achieve this objective. FDG PET/CT guided IMRT can be useful as a radiotherapy method in patients who have aggressive and non-RAI-avid but FDG-avid tumors. Further studies on this subject are needed.

## Imaging and Management with I-124 and PET

Despite the success of RAI in imaging and treatment, there are still issues in optimization of its use. The diagnostic limitations of I-131 have been described earlier. Although I-123 improves upon these drawbacks, it has limited ability to perform dosimetry, whether on a whole body or lesional basis. This, in turn, limits its ability to provide a more personalized determination of aministered activitiy for a given patient as compared to I-131. Even as a SPECT imaging agent, I-123 imaging is still limited. FDG PET imaging has superior resolution, but provides very different functional information than RAI imaging, and thus cannot supplant the latter.

Iodine-124 (I-124) is a positron emitting isotope with a half-life of approximately 4 days. This provides a high resolution RAI scan utilizing PET imaging, and its half-life enables performing dosimetry. The tracer is used for diagnostic purposes in the pre-therapy setting, however drawbacks include the complex decay schema and stunning which theoretically could be comparable to that of I-131. I-124 is more costly than I-123, and currently, the clinical availability of I-124 is limited. Nevertheless, it has been advocated as a modality that could assist in answering some critical questions regarding RAI treatment that have not been adequately addressed with other RAI agents. I-124 also has the potential to provide invaluable information in managing select individuals with high risk disease.

The superior performance of I-124 PET imaging has been validated by studies that displayed increased sensitivity compared to SPECT tracers, seeing as many as 50% more lesions in 32% more patients in the pre-therapy setting (53). A recent study by Gulec et al. (54), showed over 22% more lesions with I-124 PET/CT as compared to planar I-131 post-therapy scans. Moreover, they were able to show different iodine kinetic profiles of salivary glands, remnant thyroid tissue, and variability between various metastatic tissues.

Imaging with I-124 PET also provides an opportunity to study kinetics of in vivo processes. Freudenberg et al. (55), reported that the radiation absorbed doses using rhTSH vs. withdrawal protocols were not significantly different when patients were given the same administered activities for treatment. This finding was important because there has been a concern amongst many, when using rhTSH in the therapy of higher risk patients. Another clinically relevant use is in the protection of salivary glands from excess radiation absorbed doses. The use of agents that induce salivary secretions have been traditionally recommended. Jentzen et al. (56), proposed that the use of lemon juice immediately after I-131 therapy could increase salivary gland damage. Rosenbaum-Krumme et al. (57), showed the positive effects of rosiglitazone in a cohort of patients to improve RAI uptake in tumors with negligible or absent uptake. In a similar fashion, Ho et al. (58), showed a clinically meaningful increase in uptake in a subset of patients who underwent selumetinib therapy. The use of rhTSH in the patient preparation for RAI imaging has also been a topic of discussion.

The greatest impact of I-124 is the ability to do accurate image based lesional dosimetry to an extent that has not been possible until now. This has shown to have an impact on the management of more advanced DTC, and other issues related to RAI administration. An earlier study by Freudenberg et al. (59), showed that I-124 PET imaging had an impact on 28% of patients. The imaging not only accurately localized small lesions that were previously unsuspected, but also increased the administered I-131 activity due to lower predicted radiation doses to lesions using empiric amounts, avoided therapy administration altogether due to insufficient predicted RAI uptake, and thus led to earlier surgical management of some patients.

Despite the higher diagnostic accuracy of I-124 imaging, it is obvious that tumor biology limits even this modality. As pointed out previously, FDG is the modality that is used in diseases that have lost iodine avidity and/or in aggressive tumors. By combining the information from CT and both PET modalities (I-124 and FDG), Freudenberg et al achieved sensitivity rates of >95% for both locoregional and metastatic disease, and positive predictive values of 95-100% from just the PET information. Furthermore, they also noticed the complex behavior of tumors by noting the mixed uptake of I-124 vs. FDG (60). This has been correlated more closely with GLUT1 and Ki-67 expression by Grabellus et al (61). They demonstrated an inverse relationship between glucose and radioiodine uptake. This inverse relationship becomes more prominent as the tumors undergo dedifferentiation process.

**Figure 5:**
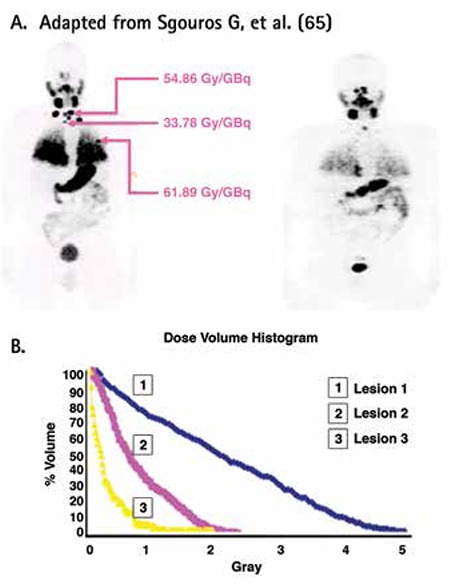
I-124 PET Pre-therapy scan (A). Serial imaging can be done to calculate lesional dosimetry as demonstrated. Even though lesional dosimetry would appear as an ideal solution, some issues remain, such as how to accurately calculate dosimetry for cases of diffuse lung uptake. Additionally, analysis of the Dose Volume Histograms of individual lesions (B) show that the uptake is quite heterogeneous so that a deeper understanding of tumor radiobiology is needed to comprehend its response, and thus pick an appropriate dosage prior to treatment. The patient was subsequently treated with 4.96 GBq I-131. A follow-up I-124 PET scan done approximately 5 months later showed a response to the degree predicted by dosimetry, but clearly disease remained despite apparent robust uptake on the prior scan. Nevertheless, the overall uptake was also decreased, suggestive of an interim therapeutic response

Although the information obtained from I-124 imaging is clearly valuable in answering some of the critical questions regarding the use of RAI, it is still unclear whether this modality is of practical use in the routine management of DTC, and the identification of patients who would benefit most from I-124 imaging remains to be further clarified. Clearly, the patients who would most likely benefit, are those with high risk disease and/or complicated medical issues. It is of no doubt that this modality can potentially contribute substantially to the understanding of the radiobiology of RAI therapy (Figure 5).

## The Follow-up Scan: The Surveillance Scan

Another controversial issue is whether to obtain a follow-up scan after a treatment cycle has been completed, during a patient’s surveillance period. This scan is typically done approximately 6-12 months after the first course of RAI treatment. The need for a surveillance scan depends upon many factors, mainly the evaluation of response to initial treatment. This can best be assessed by following thyroglobulin (Tg) levels at regular intervals on appropriate thyroxine suppression, and based on the risk stratification of the disease. Follow-up neck ultrasound results could also help guide the need for surveillance scintigraphy. If there is clinical, serologic or imaging suspicion for residual or recurrent disease in a patient initially staged as intermediate-high risk, or accurate assessment of disease status is blunted [e.g. the presence of Tg Antibodies (TgAb)] then a follow-up scan is definitely useful. In many cases, this scan may in fact serve as a pre-therapy scan as a prelude to another round of RAI treatment. The ATA guidelines give a Strong Recommendation for performing a surveillance scan in this setting (1).

The role of the surveillance scan is less clear in the low risk group, or others with no evidence of residual or recurrent disease. In the low risk group, follow-up scans confirm whether there has been successful ablation of residual thyroid tissue, or if there still is residual disease. This information is helpful in the consideration of additional RAI treatment. Even when the Tg level has not completely normalized, various groups have shown that stimulated Tg levels without imaging, or with neck ultrasounds are more sensitive in disease detection than RAI scans in low risk patients (62). It was recommended that these patients can be followed by ultrasound and Tg/TgAb in the surveillance. RAI scintigraphy should be performed only if findings warrant potential additional RAI treatment. While there is general consensus that RAI scans provide limited benefit in a low risk population, we believe that it remains important to establish a baseline. Thyroid cancer is a disease that is followed over many years, and often by several different physicians over the course of a patient’s lifetime. Thus, establishment of a baseline is helpful for long-term surveillance. The patient follow-up may be assumed by another physician and the conditions at the initial therapy could be forgotten. Additionally, in the setting of recurrence, the identification of new sites of disease could be made easier.

Another issue is that once a surveillance scan is done and is found to be negative, how often should subsequent scans be performed? As alluded to above, it may not be necessary to do more than one surveillance study in a low risk population. In fact, Caballero-Calabuig et al. (63), showed that in 10-20% of cases the RAI scan may be positive even though serum Tg levels are low or negative. This could again justify obtaining at least one RAI scan after therapy completion to document the presence of residual tissue, if any is present. However, these patients can subsequently be easily followed, with other modalities as long as there is no clinical evidence of recurrence.

Intermediate-higher risk patients have traditionally been evaluated in increasing intervals between sequential negative scans, as long as there is no evidence of disease. However, de Meer et al. (64), showed that the risk is decreased with a completely negative scan along with serologic and clinical absence of disease, and that frequent follow-up surveillance scans may not be needed as these patients appear to have similar outcomes as low risk patients. Still, the long term data, clarifying how far along the survival curve is to be extended, in the follow-up of low risk patients is not conclusive. In these patients, surveillance imaging could still be considered every 5-10 years provided that the disease status remains stable throughout the follow-up period.

**Figure 6:**
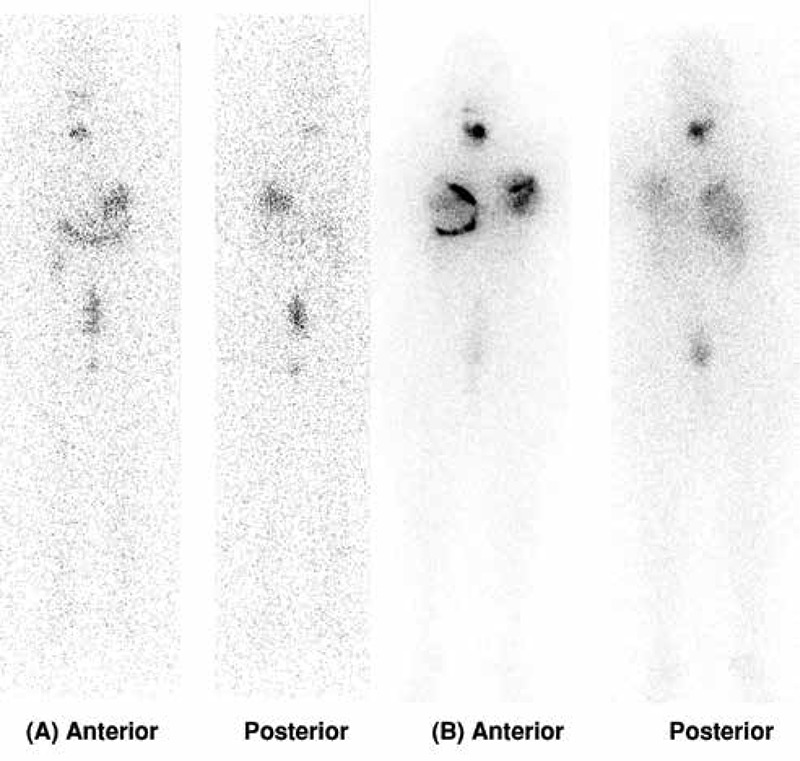
I-131 Follow-up diagnostic scan (A) 1 year after treatment with I-131 for a Low-moderate risk patient, in which a Post-therapy scan was done (B), but no pre-therapy scan. The patient had been breast feeding prior to the treatment, but was clinically thought to have stopped lactating. The post-therapy scan (B) showed bilateral diffuse breast uptake, indicating the patient’s breasts had still not returned to non-lactating state. A pre-therapy scan could have indicated this, and changed management by deferring treatment until later when lactation would have completely subsided. This is also an example of where a Follow-up scan was done after ablation. Clearly, some tissue remains in the thyroid bed, but this would not warrant further RAI treatment and could be followed. This scan can be used as a baseline. Had a scan not been done in the initial follow-up period, these findings could lead to confusion, or worse, misdiagnosis for recurrence if discovered years after the initial therapy

Using FDG PET/CT in the follow-up and surveillance setting would generally not be indicated unless there is evidence of recurrence potentially with RAI resistant disease. It can be used for follow-up in patients with known residual intermediate-high risk disease, again in whom RAI scans fail to show the disease, and in whom FDG scans have detected a lesion. There are no recommendations in how frequently these should be done, and they should be performed based on the stability of the patient’s clinical course (Figure 6).

## Future Goals and Directions

In the final analysis, the use of all diagnostic tools can only be as good as the user who understands their value, and the tools should not be restricted but rather balanced between their cost and impact on improving overall patient care. The understanding of disease biology as well as the strengths and limitations of the diagnostic tools continue to evolve. More comprehensive information will help refine the treatment of DTC, and may actually set a balance between limiting potentially unnecessary radiation and delivering appropriate radiation doses to achieve a result that is more therapeutic than futile.

The use of RAI and FDG also evolve parallel to the increased sophistication in treatment methods. As discussed, I-124 can be used to refine the utilization of RAI therapy in cases when it could be avoided, or pursuing aggressive treatment when the study shows it to be feasible. Since the limitations associated with RAI are becoming better understood, it is likely that future treatments will involve combining RAI with other modalities, allowing for more thorough evaluations. FDG may be used to identify patients who are RAI refractory prior to starting treatment, and thus both RAI and targeted therapy could be used to treat different aspects of the same disease. The enhanced information provided by I-124 PET/CT can be coordinated with external beam treatments in advanced disease to target radiation to certain areas of the tumor, whereas FDG could be used to provide an idea where the external beam radiation should be intensified.

## Conclusion

The use of theranostic molecular imaging is very valuable if used to its full capacity. It is important to realize that the sophisticated techniques work best when used in the proper clinical context, after evaluating the advantages vs. disadvantages of any intervention. As stated earlier, the utility of imaging will likely evolve, and the recommended protocols will require adaption to changing paradigms. Some applications may only be accessible to skilled surgeons in high volume centers, endocrinologists experienced in the follow-up of patients, or oncologists well versed with the treatment of advanced disease. Hence, the use of these techniques and technologies could vary between high volume academic centers, where more advanced disease tends to be seen, versus other practices where technical and expert personnel resources may be limited. Additionally, it is important to use all the information available to determine when and how to perform imaging. The clinical management decisions should definitely incorporate Tg, neck ultrasounds, as well as clinical assessment of the patient, including staging and subsequent clinical and molecular risk stratification.

## Figures and Tables

**Table 1:**
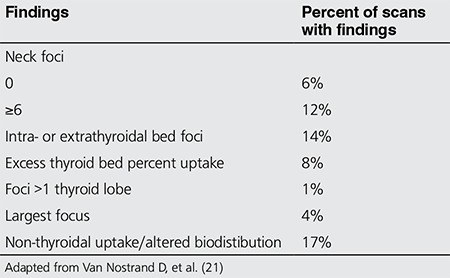
Pre-therapy scanning
